# Potential Development Ability of Residual Zoites, a Second-Generation Meront, Inducing Long-Term Infection by the Mouse Eimerian Parasite, *Eimeria krijgsmanni*

**DOI:** 10.1007/s11686-024-00910-2

**Published:** 2024-08-29

**Authors:** Masanobu Mizuno, Satoru Kiyotake, Makoto Matsubayashi, Takane Kaneko, Hitoshi Hatai, Yoshikazu Fujimoto, Moe Ijiri, Hiroaki Kawaguchi, Toshihiro Matsui, Tomohide Matsuo

**Affiliations:** 1 Laboratory of Parasitology, Joint Faculty of Veterinary Medicine, Kagoshima University, Kagoshima, 890-0065 Japan; 2 Laboratory of Veterinary Immunology, Graduate School of Veterinary Science, Osaka Metropolitan University, Izumisano, 598-5831 Osaka Japan; 3 Department of Life Sciences, Faculty of Life Sciences, Kyushu Sangyo University, Fukuoka, 813-8503 Japan; 4 Farm Animal Clinical Skills and Diseases Control Center, Iwate University, Morioka, 020-8550 Iwate Japan; 5 Transboundary Animal Diseases Research Center, Joint Faculty of Veterinary Medicine, Kagoshima University, Kagoshima, 890-0065 Japan; 6 Laboratory of Veterinary Pathology, School of Veterinary Medicine, Kitasato University, Towada, 034-8628 Aomori Japan; 7 Seisen University, Higashi Gotanda, Tokyo, 141-8642 Japan

**Keywords:** *Eimeria*, Mouse, Immunosuppression, Immunodeficiency, Latent infection

## Abstract

**Purpose:**

Coccidiosis caused by eimerian parasites results in lethal watery or bloody diarrhea in hosts, and markedly impairs the growth of and feed utilization by host animals. We previously investigated detailed the life cycle of *Eimeria krijgsmanni* as a mouse eimerian parasite. Only second-generation meronts, as an asexual stage, were morphologically detected in the epithelium of the host cecum for at least 8 weeks after infection, even though oocyst shedding finished approximately 3 weeks after infection. The presence of zoites was of interest because infection by eimerian parasites is considered to be self-limited after their patent period.

**Methods:**

To clarify the significance of residual second-generation meronts in *E*. *krijgsmanni* infection, we performed infection experiments using immunocompetent mice under artificial immunosuppression and congenital immunodeficient mice.

**Results:**

The number of oocysts discharged and the duration of oocyst discharge both increased in immunosuppressed mice. In immunodeficient mice, numerous oocysts were shed over a markedly longer period, and oocyst discharge did not finish until 56 days after inoculation.

**Conclusions:**

The present results suggest that the second-generation meronts survived in the epithelial cells of the cecum after the patent period, thereby contributing to extended infection as an asexual stage. The results obtained on *E*. *krijgsmanni* indicate that infections by *Eimeria* spp. are not self-limited and potentially continue for a long period of time.

## Introduction

*Eimeria* spp., belonging to the phylum Apicomplexa, are the intracellular protozoan parasites, that are found in most classes of vertebrates worldwide [1] and exhibit high host specificity. Coccidiosis caused by eimerian parasites results in lethal watery or bloody diarrhea in hosts, and markedly impairs the growth of and feed utilization by host animals. Although drugs and vaccines are available for some species [2, 3], a number of limitations, such as safety issues, troublesome vaccine strategies, and the emergence of drug resistant strains have a negative impact on their effectiveness [4, 5]. Therefore, further research is needed to develop new methods for the prevention and/or treatment of infection by *Eimeria* spp. However, experiments on eimerian parasites may be technically challenging. Well-established methods to replicate life cycles using in vitro cultures, such as those for *Toxoplasma gondii* and *Babesia* spp., are not yet available for *Eimeria* spp. [6], and infection experiments using large animals, including cattle, are also challenging. Therefore, we hypothesized that infection experiments using mice may be useful to efficiently study on eimerian parasites.

Fourteen species of murine *Eimeria* have been described to date [7, 8], and a number of studies have been published on *Eimeria falciformis*, *E*. *papillata*, and *E*. *vermiformis*. However, murine *Eimeria* infection remain unclear. We previously reported our findings on the host specificity [9], life cycle [10], and drug susceptibility [11, 12] of *E*. *krijgsmanni* as a mouse eimerian parasite. The life cycle of *Eimeria* spp. consists of asexual and sexual developmental stages and is self-limited, which means that parasites disappear and the host completely recovers from infection after the patent period finishes. In *E*. *krijgsmanni*, asexual stages followed by sexual stages involve four generations, and oocyst shedding finishes approximately 3 weeks after infection [10]. Furthermore, the host acquires resistance to subsequent infection at least 3 weeks after the primary infection [11]. We previously showed that only second-generation meronts (meront II), an asexual stage in the host, were morphologically detected in the epithelium of the cecum at least until 8 weeks after infection [10]. This was the first study to show the presence of special zoites detected for a long time after the patent period of infection by *Eimeria* spp.

The present study investigated the significance of meront II in infection by *E*. *krijgsmanni* because our previous findings were based solely on morphological observations and, thus, it was unclear whether residual meront II were alive. We herein conducted infection experiments using immunocompetent mice under artificial immunosuppression. Additionally, congenital immunodepression model mice, namely, severe combined immunodeficiency (SCID) mice and nude mice, were used in infection experiments to investigate *E*. *krijgsmanni* infection in the absence of host immune responses.

## Materials and Methods

### Mice

Five-week-old female immunocompetent (BALB/c) and immunodeficient (SCID and nude) mice (CREA Japan, Tokyo) were used in each experiment. All mice were confirmed by a fecal examination to be free of natural infection by *Eimeria* spp., and were raised under coccidian-free conditions until used in experiments.

### Parasites

*E*. *krijgsmanni* was obtained from the Division of Tropical Diseases and Parasitology, Department of Infectious Diseases, Kyorin University School of Medicine, and maintained by routine passage through ICR mice (CLEA Japan, Tokyo) at the Laboratory of Parasitology, Joint Faculty of Veterinary Medicine, Kagoshima University. Oocysts were collected from the feces of infected mice and stored at 4 °C until used after sporulation in 2% potassium dichromate solution at 25 °C for several days.

### Infection and Assessment of Infection

All mice were orally inoculated with 2.0 × 10^2^ oocysts. The discharge of oocysts in the feces was examined using the sugar flotation method. If positive, the number of oocysts per gram of feces (OPG value) was estimated [9]. In all infection experiments, feces collected from 5 mice in each group were checked daily. OPG values were shown as the average of 5 mice in the present study.

### Artificial Immunosuppression

Dexamethasone (DXM) (SIGMA, Tokyo) suspended in sterilized water was prepared at 1 mg/ml and administered at 100 µl/mouse (0.1 mg/mouse) daily via subcutaneous injection. The dose was estimated according to the average dose for general immunosuppression treatment because of the wide range of DXM doses reported in various studies [13–15]. Twenty mice treated with DXM were divided into 4 groups (5 mice each) according to treatment schedules (Table 1): from 2 days before infection to 15 days post-infection (PI) (− 2–15 DXM), from 2 days before infection to 21 days PI (− 2–21 DXM), from 2 days before infection to 56 days PI (− 2–56 DXM), from 15 to 56 days PI (15–56 DXM), and from 21 to 56 days PI (21–56 DXM). These administration periods were selected for the following reasons: the presence of meront II may be detected in the epithelium of the cecum for at least 56 days PI [10], the first oocyst shedding period was completed at 15 days PI, and immunocompetent mice exhibited resistance to challenge infection at 21 days PI [9]. An additional control group (5 mice) treated with 100 µl of sterilized distilled water (DW) via subcutaneous injection from 2 days before infection to 56 days PI was also established. In addition, a group of mice was treated with DMX from 28 days to 41 days PI (28–41 DXM) by the administration of 100 µl/mouse (1 mg/mouse) daily via subcutaneous injection. The patent period finished and hosts acquired immunity against *E*. *krijgsmanni* until 21 days PI. Therefore, on day 28, residual meront II had not been able to develop to the next stage in resistant hosts. The administration dose and schedule for the 28-41DXM group were selected based on the results of preliminary experiments in which administration at 0.1 mg/mouse did not induce the restart of oocyst shedding (data not shown), and treatment was concluded after 2 weeks to minimize the risk of adverse health effects related to the relatively high dose of DXM.

**Table 1:**
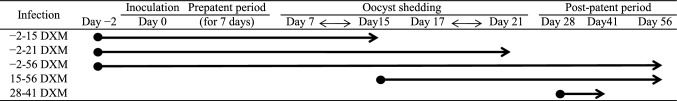
Schedules of dexamethasone treatments

Oocyst-shedding periods were divided in immunocompetent mice

Day 15, end of the first oocyst shedding period; Day 21, end of the second oocyst shedding period

Second-generation meronts were detected in the epithelium of the cecum until Day 56

Two immunodeficient mouse groups (SCID and nude; 5 mice each) were also prepared to examine their oocyst shedding in the infection without host immunity.

### Ethics

Animal experiments in the present study were performed with the approval of the Committee for Animal Care and Use of Kagoshima University (VM17053/21053). Laboratory animal care and use protocols adhered to the AAALAC international approved program of the Experimental Animal Center, Kagoshima University.

## Results

**Fig. 1:**
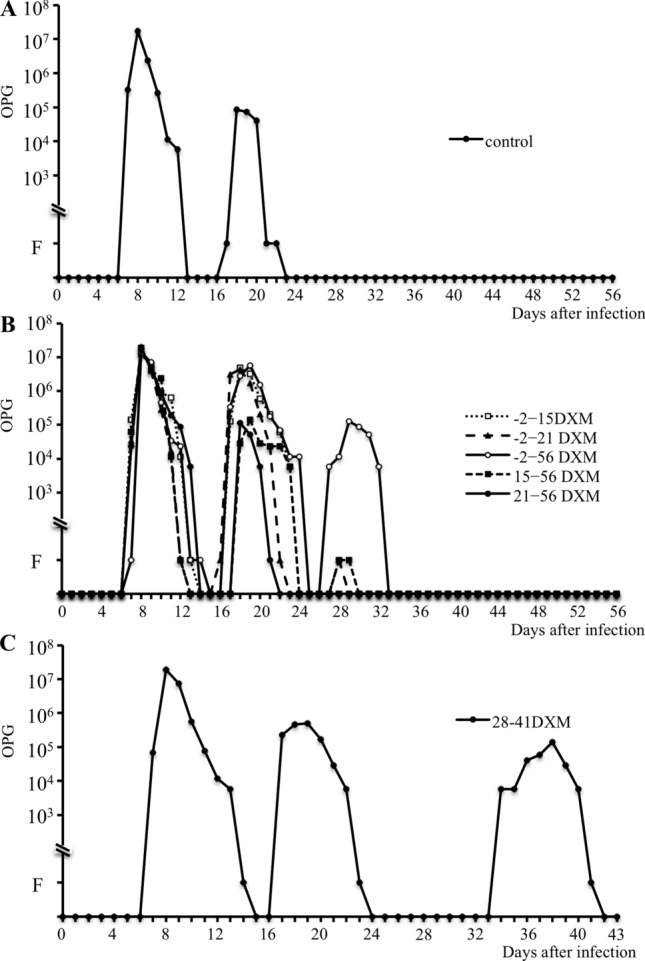
OPG values in experimental groups treated with DW (**a**) and DXM (**b** and **c**) with primary infection. **a** Divided oocyst-shedding periods lasted until approximately 3 weeks after infection. **b** OPG values in the − 2–15, − 2–21, − 2–56, 15–56 and 21–56 DXM groups. The number of oocysts shed and the duration of shedding were higher and longer, respectively, in each DXM group than in the DW group. **c** OPG value in the 28-42DXM group. Oocyst discharge clearly restarted 7 days after the start of DXM administration and continued for 8 days. F: Positive for oocysts by the sugar flotation method

The oocyst-shedding pattern in the DW group and each DXM experimental group is shown in Fig. 1. Two peaks in OPG values were confirmed in the DW group (Fig. 1A). In the DXM groups, the oocyst-shedding pattern slightly changed with the duration of treatment (Fig. 1B). Two peaks in OPG values were observed in the − 2–15 and 21–56 DXM groups, and three peaks in the − 2–21, − 2–56 and 15–56 DXM groups. The maximum OPG value and the duration of the second peak in the DW and 21–56 DXM groups not yet treated with DXM were very similar. However, the second peak in the − 2–15, − 2–56 and 15–56 DXM groups was prolonged, and the maximum OPG value was higher in the − 2–15, − 2–21, and − 2–56 DXM groups than in the other groups. A third peak in the OPG value was confirmed in the − 2–21, − 2–56 and 15–56 DXM groups; however, the number of shed oocysts markedly differed. A small number of oocysts were shed on days 1 and 2 in the − 2–21 and 15–56 DXM groups, respectively, whereas oocysts were shed for 6 days in the − 2–56 DXM group, and the maximum OPG value reached 10^5^. The number of shed oocysts increased and/or the patent period was prolonged in each experimental group, although differences among these experimental groups were not significant. Oocyst shedding in the 28–42 DXM group, in which treatment was withheld until 28 days PI, stopped 24 days PI; however, shedding restarted 7 days after the beginning of treatment on day 28 (Fig. 1C). No particular health issues occurred in any of the mice in the DXM groups.

**Fig. 2:**
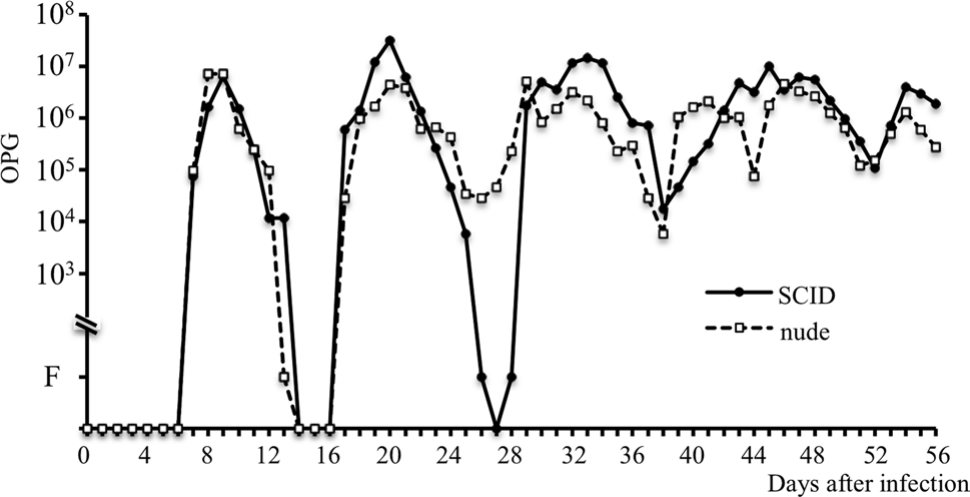
OPG values in immunodeficient mice (SCID and nude) with primary infection. Oocyst discharge did not finish until 56 days after the primary inoculation. The number of shed oocysts significantly increased and decreased with a periodicity of approximately 7 days. OPG: oocysts per gram of feces. F: Positive for oocysts by the sugar flotation method

The oocyst-shedding patterns of the SCID and nude mice groups with primary infection are shown in Fig. 2. A larger number of oocysts were shed over a very long period, which did not finish until 56 days PI in these immunodeficient mouse groups. Additionally, the OPG values meaningfully fluctuated with a periodicity of approximately 7 days.

## Discussion

Infections with *Eimeria* spp. are generally considered to be ‘self-limited’ with recovery occurring at the end of oocyst shedding. In our previous studies on *E*. *krijgsmanni*, we also considered the patent period to have a single peak [9, 12]. However, in the present study, the oocyst-shedding pattern was investigated for a long time, and the oocyst-shedding period always exhibited 2 peaks separated by approximately 3 weeks after inoculation in immunocompetent mice. Furthermore, Ono *et al*. [10] morphologically demonstrated that only meront II were present in the epithelial cells of the cecum, in which *E*. *krijgsmanni* normally develop, for up to 56 days PI after the end of oocyst shedding. Only meront II appeared to be present in the cecum for a short time before and after day 14, and third- and fourth-generation meronts and sexual developmental stages emerge again according to histological observations (unpublished data). These results suggest that the life cycle temporarily stopped and then restarted from the meront II stage. In addition, immunocompetent mice appeared to acquire resistance against parasites until 3 weeks PI and development from meront II to third-generation meronts was suppressed after the second peak in the OPG value.

The number of oocysts was higher and the shedding period was longer in the DXM groups than in the control group although the number of shed oocysts varied among the groups treated with different DXM doses and schedules. Additionally, a clear restart of oocyst discharge was confirmed in the 28–41 DXM group indicating that surviving residual meront II in resistant hosts developed again with the immunosuppression treatment and oocyst discharge was led by third- and fourth-generation meronts and sexual reproductive stages. This result suggests that the development of meront II was reactivated by immunosuppression. Among other murine *Eimeria* spp., such as *E*. *vermiformis* and *E*. *falciformis* var. *pragensis*, resistance against challenge infection 20 and 25 days after the primary infection, respectively, has been reported [16, 17]. Furthermore, immunocompetent mice showed resistance to challenge infection 21 days after the primary infection with *E. krijgsmanni* [9]. Therefore, meront II appear to survive in the epithelial cells of the cecum in resistant hosts and contribute to latent infection unknown in other *Eimeria* spp.

In immunodeficient model mice (SCID and nude), numerous oocysts were discharged and the transition in their OPG values significantly fluctuated approximately every week. Nude mice are athymic mice with congenital T cell deficiencies, whereas SCID mice are deficient in T and B cells [18–20]. Differences in the immunological status between SCID and nude mice did not appear to have a marked impact on infection by *E*. *krijgsmanni*. These phenomena in immunodeficient hosts have not been reported for eimerian parasites. Although *E*. *krijgsmanni* may continue to discharge numerous oocysts for a long time, eimerian parasites cannot cause self-infection, such as that by *Cryptosporidium* parasites. The number of generations in asexual reproduction is species-specific, but there may be unknown developmental stages as potential mechanisms that enable extended infections with the discharge of numerous oocysts.

The present results on *E*. *krijgsmanni* indicate that infections with some *Eimeria* spp. are not temporary or self-limited. The presence of meront II and the restarting of oocyst shedding in immunosuppressed hosts were observed. For example, *Toxoplasma gondii*, a coccidian parasite, causes long-lasting infections in intermediate hosts. Tachyzoites of *T*. *gondii* transform into bradyzoites and form cysts to escape host immune responses and continue infection only in particular organs, such as the muscles and brain, where host immune response are minimal. In contrast, in the present study, meront II, which are a normal developmental stage, remained in the epithelium of the cecum, the normal site of parasitism. Therefore, meront II may represent a special developmental stage capable of tactically avoiding and/or resisting host immunity. The bradyzoites of *T*. *gondii* may also be reactivated by host immunosuppression [21, 22]. Therefore, meront II of *E*. *krijgsmanni* may be regarded as the cyst-like asexual stage. Furthermore, an approximately 7-day cycle was observed in the oocyst-shedding pattern of both immunocompetent and immunodeficient mice: the patent period of immunocompetent mice had two peaks at approximately 7 days each, and the number of shed oocysts in immunodeficient mice increased and decreased with a periodicity of approximately 7 days. In immunocompetent mice, parasites may not be able to develop to third-generation meronts due to acquired immunity. Some meront II continue to develop while others rest in repeated cycles. The present results also suggest that *E*. *krijgsmanni* exhibits an independent developmental rhythm for the extension of infection because host immunity is unlikely to change every 7 days. However, in the present study, infection caused by at least some eimerian parasites may be extended over a long period. Further investigations on meront II of *E. krijgsmanni* are needed to clarify the mechanisms underlying latent infection by coccidian parasites.

## Conclusion

The presence of *E*. *krijgsmanni* second-generation meronts in the host intestinal epithelium was confirmed in our previous study, even though infections with eimerian parasites are known to be self-limited until the patent period. However, our previous findings were based solely on morphological observations. The present study employed infection experiments using immunocompetent mice under experimental immunosuppression and mice with inborn immunodeficiency. The results obtained that second-generation meronts survived and contributed to unknown latent infections.
